# Correction to [Ulcerative colitis associated mononeuritis multiplex in 75‐year‐old patient: Rare case report]

**DOI:** 10.1002/ccr3.9409

**Published:** 2024-09-04

[Pourbagherian O, Jafarpour M, Sepideh Tahsini Tekantapeh, Eftekharsadat AT, Javid M. Ulcerative colitis associated mononeuritis multiplex in 75‐year‐old patient: Rare case report. Clin Case Rep. 2024 Jul 4;12(7):e9136. doi: 10.1002/ccr3.9136. PMID: 38966290; PMCID: PMC11222965.]

Full name correction:

In the published article titled “Ulcerative colitis associated mononeuritis multiplex in 75‐year‐old patient: Rare case report,” an author's name was mistakenly formatted as Sepideh Tahsini. The author's name should be formatted as Sepideh Tahsini Tekantapeh.

Please correct the suffix of my name in the list of authors as:

Sepideh Tahsini Tekantapeh

We apologize for this error.

## AUTHOR CONTRIBUTIONS

**Omid Pourbagherian:** Methodology; validation; writing – original draft. **Mehdi Jafarpour:** Supervision; validation. **Sepideh Tahsini Tekantapeh:** Formal analysis; resources; software. **Amirtaher Eftekharsadat:** Data curation; resources. **Maryam Javid:** Formal analysis; resources; writing – original draft.

